# Model-Based Evaluation of Closed-Loop Deep Brain Stimulation Controller to Adapt to Dynamic Changes in Reference Signal

**DOI:** 10.3389/fnins.2019.00956

**Published:** 2019-09-10

**Authors:** Fei Su, Karthik Kumaravelu, Jiang Wang, Warren M. Grill

**Affiliations:** ^1^Department of Biomedical Engineering, Duke University, Durham, NC, United States; ^2^School of Mechanical and Electrical Engineering, Shandong Agricultural University, Tai'an, China; ^3^School of Electrical and Information Engineering, Tianjin University, Tianjin, China

**Keywords:** closed-loop deep brain stimulation, Parkinson's disease, beta band activity, proportional-integral controller, Routh-Hurwitz stability analysis

## Abstract

High-frequency deep brain stimulation (DBS) of the subthalamic nucleus (STN) is effective in suppressing the motor symptoms of Parkinson's disease (PD). Current clinically-deployed DBS technology operates in an open-loop fashion, i.e., fixed parameter high-frequency stimulation is delivered continuously, invariant to the needs or status of the patient. This poses two major challenges: (1) depletion of the stimulator battery due to the energy demands of continuous high-frequency stimulation, (2) high-frequency stimulation-induced side-effects. Closed-loop deep brain stimulation (CL DBS) may be effective in suppressing parkinsonian symptoms with stimulation parameters that require less energy and evoke fewer side effects than open loop DBS. However, the design of CL DBS comes with several challenges including the selection of an appropriate biomarker reflecting the symptoms of PD, setting a suitable reference signal, and implementing a controller to adapt to dynamic changes in the reference signal. Dynamic changes in beta oscillatory activity occur during the course of voluntary movement, and thus there may be a performance advantage to tracking such dynamic activity. We addressed these challenges by studying the performance of a closed-loop controller using a biophysically-based network model of the basal ganglia. The model-based evaluation consisted of two parts: (1) we implemented a Proportional-Integral (PI) controller to compute optimal DBS frequencies based on the magnitude of a dynamic reference signal, the oscillatory power in the beta band (13–35 Hz) recorded from model globus pallidus internus (GPi) neurons. (2) We coupled a linear auto-regressive model based mapping function with the Routh-Hurwitz stability analysis method to compute the parameters of the PI controller to track dynamic changes in the reference signal. The simulation results demonstrated successful tracking of both constant and dynamic beta oscillatory activity by the PI controller, and the PI controller followed dynamic changes in the reference signal, something that cannot be accomplished by constant open-loop DBS.

## Introduction

Parkinson's disease (PD) is characterized by degeneration of dopaminergic neurons in the substania nigra pars compacta (SNc) resulting in motor symptoms including bradykinesia, rest tremor, postural instability, and rigidity (Davie, 2008; Jankovic, 2008). High-frequency deep brain stimulation (DBS) of the subthalamic nucleus (STN) or globus pallidus internus (GPi) is a well-established surgical therapy to treat the motor symptoms of PD (Krack et al., 2003; Rodriguez-Oroz et al., 2005; Odekerken et al., 2016). Current clinical DBS technology is open loop—stimulation is always on and the stimulation parameters are tuned periodically through manual adjustments by health care professionals. The process of selection of DBS parameters is challenging due to the large number of parameters (Kuncel and Grill, 2004). Therefore, the efficacy of current open-loop DBS may be suboptimal and patients can experience side effects, including speech deficits and cognitive dysfunction (Deuschl et al., 2006; Okun and Foote, 2010; Massano and Garrett, 2012; Cyron, 2016).

Recent clinical studies suggest that closed-loop DBS (CL DBS) may be more efficient at suppressing PD motor symptoms with reduced side effects as compared to continuous high-frequency STN DBS (Rosin et al., 2011; Carron et al., 2013; Hebb et al., 2014; Rossi et al., 2016). However, the design of CL DBS controllers comes with several challenges including selection of a feedback signal reflecting PD symptoms and the capacity of the controller to adapt to dynamic changes in the reference signal (Hebb et al., 2014; Arlotti et al., 2016a; Parastarfeizabadi and Kouzani, 2017). Concurrent neuronal recordings and behavioral assessments from PD patients and animal models of PD showed a strong correlation between beta band oscillations (13–35 Hz) and PD motor symptoms, especially bradykinesia (Zaidel et al., 2010; Jenkinson and Brown, 2011; Little and Brown, 2012; Hoang et al., 2017), and beta band activity may be an appropriate feedback signal for CL DBS. However, beta oscillations in the basal ganglia desynchronize in preparation and during voluntary movement (Levy et al., 2002; Brittain and Brown, 2014). Therefore, a fixed beta power reference may not be appropriate for control of DBS, and it may be beneficial to include in the controller design the ability to adapt to dynamic changes in the reference signal.

The objective of this study was to design a controller for CL DBS that can adapt to dynamic changes in the reference signal. We evaluated the performance of a proportional integral (PI) controller using a network model of the basal ganglia (BG) (Kumaravelu et al., 2016). The parameters of the PI controller were tuned by coupling a linear controlled auto-regressive (CAR) model with Routh-Hurwitz stability analysis. The PI controller was successful in adapting to dynamic changes in the reference signal, and such a control scheme may be suitable for implementation in CL DBS systems.

## Methods

**Figure 1:**
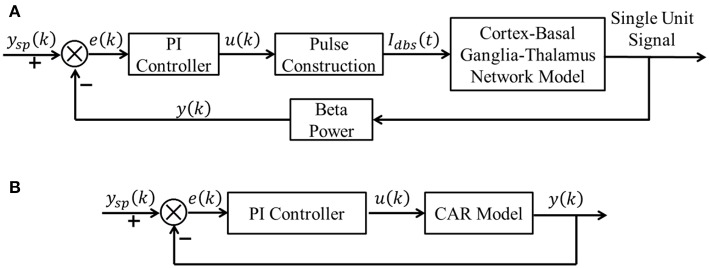
**(A)** The CL DBS framework. The spike times of model neurons in the GPi were calculated, and the beta band power of these spike times was used as the feedback signal *y* (*k*). The error term *e* (*k*) between the desired beta power *y*_*sp*_ (*k*) and actual value *y* (*k*) was input to the PI controller to calculate the stimulation frequency *u* (*k*). The stimulation signal *I*_*dbs*_ (*t*) delivered to the cortex-basal ganglia-thalamus network model was subsequently determined. **(B)** The transformed linear system of the CL DBS system. This transformed linear system was used to determine the appropriate parameters for the PI controller, and the PI parameters were constant once calculated.

A block diagram of the proposed CL DBS framework is shown in Figure 1A. The signal power of model neuron activity in the beta band was used as the feedback signal *y*(*k*), and the error *e*(*k*) between the actual beta power and the desired beta power *y*_*sp*_(*k*) was sent to the PI controller to calculate the stimulation frequency *u*(*k*). Thus, the PI controller calculated the DBS frequency according to the variation of beta oscillatory power. The calculated DBS frequency determined the time of the next stimulation pulse *I*_*dbs*_(*t*) delivered to a biophysical network model of the parkinsonian cortex-basal ganglia-thalamus (CTx-BG-Th) network. The selection of appropriate PI controller parameters was required for the actual beta power to track dynamic variations in the desired power. Below we propose a stability analysis method to calculate automatically the PI parameters.

### Computational Model of the Cortex-Basal Ganglia-Thalamus Network

We used a model of the CTx-BG-Th network as a test bed to evaluate the performance of the closed-loop control scheme (Kumaravelu et al., 2016), and a implementation of this model in MATLAB can be downloaded from ModelDB (https://senselab.med.yale.edu/modeldb/). The CTx-BG-Th model included the cortex, striatum, STN, globus pallidus externus (GPe), GPi and a thalamic nucleus, and each region was comprised of 10 single-compartment Hodgkin-Huxley type neurons. In the original publication, the model was validated extensively, including matching the responses evoked in the basal ganglia by cortical stimulation in rats (Kita and Kita, 2011), model neuron firing rates and patterns that were consistent with parkinsonian rats (Mallet et al., 2008), and responses to STN DBS at different frequencies that matched those measured experimentally (McConnell et al., 2012; So et al., 2012). Model BG neurons exhibited exaggerated low-frequency oscillatory activity in the parkinsonian state compared to the healthy condition, similar to that seen *in vivo*. Since, beta oscillatory activity is well-correlated with PD symptoms (Leventhal et al., 2012; Stein and Bar-Gad, 2013), we chose the beta band (13–35 Hz) power present in the activity of the GPi neurons as the model-based proxy for symptoms (Brocker et al., 2013) to evaluate the effectiveness of the CL DBS controller. There is a strong correlation between single unit firing and LFPs in the beta band in the STN (Levy et al., 2002; Kühn et al., 2005) and in the GP (Goldberg et al., 2004), and the power spectrum calculated from the single unit spike times of GP neurons was correlated with motor symptoms of parkinsonism (McConnell et al., 2012). Simulations were implemented in MATLAB R2016a and equations were solved using the forward Euler method with a time step of 0.01 ms; spectral analyses were performed using the “mtspecgrampt” function of the Chronux neural signal analysis package (chronux.org) (sliding 1 s window, 0.1 s step size and [3 5] tapers (3 is the time-bandwidth product and 5 is the number of tapers)). The spectrum of all 10 GPi neurons spike time series was calculated using the multi-taper spectral estimation method.

### Identification of Relationship Between Stimulation Frequency and Beta Band Power of GPi Model Neurons Spike Times

The oscillations within the CTx-BG-Th network were similar across the different parts of the loop (Kumaravelu et al., 2016), for STN, GPi, and GPe both single neuron and local field potentials (LFPs) exhibited excessive beta band oscillation in the PD state, while for thalamus and cortex single neuron oscillation were not dominant (Stein and Bar-Gad, 2013). The beta band power of GPi model neurons spike times was chosen to characterize the model state. The dynamics of the CTx-BG-Th network were highly non-linear and therefore it was inappropriate to use the linear PI controller to control directly the network model of PD. A linear model of the plant between the stimulation frequency and the beta band power of GPi model neuron spike times was first identified using a CAR model. The structure of a CAR model was

(1) $$\begin{array}{l} \left(1+a_{1}z^{-1}+a_{2}z^{-2}+\cdots +a_{n_{a}}z^{-n_{a}}\right)y\left(k\right)\\ =\left(b_{0}+b_{1}z^{-1}+b_{2}z^{-2}+\cdots +b_{n_{b}}z^{-n_{b}}\right)u\left(k\right)+\varepsilon \left(k\right) \end{array}$$

where z was the lag operator, *u*(*k*) was the input signal (stimulation frequency) and *y*(*k*) was the output signal (beta power of GPi model neuron spike times), *n*_*b*_ and *n*_*a*_ were the order of input and output sequences, respectively, and ε(*k*) was assumed to be white noise. The identification process included the following steps:

1. Collect input and output data from the CTx-BG-Th network model.
2. Estimate model parameters *a*_1_ ⋯ *a*_*n*_*a*__ and *b*_0_ ⋯ *b*_*n*_*b*__.
3. Choose appropriate order parameters *n*_*a*_ and *n*_*b*_.
4. Quantify the prediction accuracy of the CAR model.

**Figure 2:**
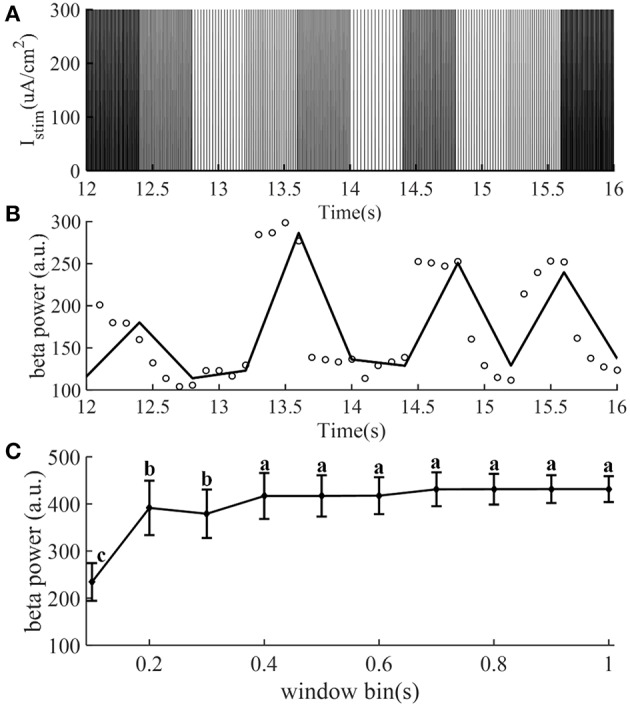
The stimulation sequence **(A)** and beta band power **(B)** obtained from the CTx-BG-Th network model to train the CAR model. Only data from 12 to 16 s are presented to improve visualization. **(A)** The stimulation frequency was randomly selected from 5 to 200 Hz, and for each frequency the corresponding stimulation sequence lasted for 0.4 s. **(B)** Circles represent the beta power value in each 0.1 s, the collected beta power within 0.4 s was the average of four values. **(C)** Mean ± standard deviation of beta power of GPi model neuron spike times plotted as a function of time window bin (50 trials). The mean value of beta power varies across different time window bin values, and values not sharing the same letter were significantly different (*p* < 0.05, Tukey's HSD).

The identification accuracy of the CAR model was highly dependent on the input output data that were selected, because not all data provided an equal amount of information (Ljung, 1999). The designed stimulation sequence was delivered to the CTx-BG-Th model (in the open loop), and the corresponding output data (beta band power) was calculated. To obtain more informative input/output data to identify the CAR model, the frequencies (input data) of the stimulation waveform were chosen randomly between 5 and 200 Hz. Figure 2A illustrates the stimulation sequence from 12 to 16 s, illustrating that each frequency continued for 0.4 s to ensure that at least two pulses were delivered for each random frequency. The simulation duration was 400 s, resulting in responses to 1,000 frequency samples. The stimulation sequence was delivered to the computational model of the CTx-BG-Th network, and spiking activity was recorded from GPi model neurons. The time window used to bin the beta power of GPi spike times was sensitive to the temporal dynamics of beta power when the stimulation frequencies were randomly changed (Figure 2C). Differences in beta power across time window bins were compared using one-way ANOVA with *post-hoc* Tukey's honestly significant difference (HSD) test, and statistical significance was defined as α = 0.05. The beta power varied across different time window bins (F = 252.54, *p* < 0.0001). When the time window bin was larger than 0.1 s, the calculated beta power was no <1.6 times the value with time window bin equaled to 0.1 s. The choice of the short 0.1 s bin enabled capture of small dynamic changes in beta power, as our objective was to implement a controller that responded to such changes. Bin sizes of 0.2 s or longer did not reflect the dynamic variation of the beta power, as indicated by the invariance to bin size. Since each frequency was delivered for 0.4 s and the bin used to calculate beta power was 0.1 s, the beta power obtained in Figure 2B was the average of four values within 0.4 s.

We used the recursive least squares (RLS) method (Ljung, 1999) to estimate the CAR model parameters. The CAR model was transformed into a standard LS form (Ljung, 1999),

(2) $$\begin{array}{l} y\left(k\right)=-a_{1}y\left(k-1\right)-a_{2}y\left(k-2\right)-\cdots -a_{n_{a}}y\left(k-n_{a}\right)\\ \;+b_{0}u\left(k\right)+b_{1}u\left(k-1\right)+b_{2}u\left(k-2\right)+\cdots \\ \;+b_{n_{b}}u\left(k-n_{b}\right)+\varepsilon \left(k\right)=\varphi^{T}\left(k\right)\theta +\varepsilon \left(k\right) \end{array}$$

where $\varphi \left(k\right)=[-y\left(k-1\right),\cdots ,-y\left(k-n_{a}\right),u\left(k\right),\cdots ,u(k-{n_{b})]}^{T}$ was the known sequence of input and output data, and $\theta {=\left[a_{1},a_{2},\cdots ,a_{n_{a}},b_{0},b_{1},\cdots ,b_{n_{b}}\right]}^{T}$ was the vector of unknown model parameters. From Equation (2), the current value of the output signal was correlated with the past input and output signals as well as the current input signal. Then, unknown model parameters were estimated by the RLS method,

(3) $$\begin{array}{l} y_{e}\left(k\right)=\varphi^{T}\left(k\right)\hat{\theta } \end{array}$$

where $\hat{\theta }$ was the estimated parameter vector calculated using the following equations:

(4) $$\{\begin{array}{c} \hat{\theta }\left(k\right)=\hat{\theta }\left(k-1\right)+K\left(k\right)\left[y\left(k\right)-\varphi^{T}\left(k\right)\hat{\theta }\left(k-1\right)\right]\\ K\left(k\right)=\frac{P\left(k-1\right)\varphi \left(k\right)}{1+\varphi^{T}\left(k\right)P\left(k-1\right)\varphi \left(k\right)}\\ \;P\left(k\right)=\left[I-K\left(k\right)\varphi^{T}\left(k\right)\right]P\left(k-1\right) \end{array}$$

The root mean square error (RMSE) between the actual output signal and the CAR model predicted output signal was used to quantify the prediction accuracy of the CAR model,

(5) $$\begin{array}{l} e_{RMSE}=\sqrt{\frac{1}{N}\sum_{k=1}^{N}{\left(y\left(k\right)-y_{e}\left(k\right)\right)}^{2}} \end{array}$$

**Figure 3:**
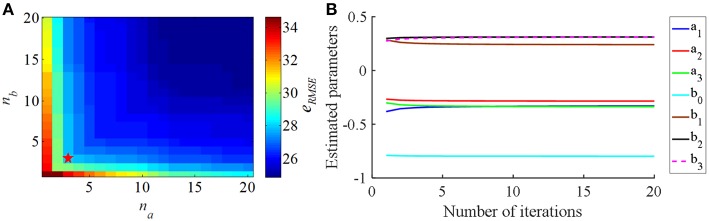
**(A)** The relationship between the CAR model order parameters (*n*_*a*_ and *n*_*b*_) and the RMS error (*e*_RMSE_) between the actual output signal and the CAR model predicted output signal. **(B)** The estimated CAR model parameters across iterations to minimize *e*_RMSE_.

The *e*_*RMSE*_ declined as the CAR model order (*n*_*a*_ and *n*_*b*_) was increased (Figure 3A). Since the purpose of the identified CAR model was to design the PI controller but not to substitute for the original CTx-BG-Th network model, we were not interested in higher-order dynamics. Akaike's information criterion (AIC) was used to select the model order (McQuarrie and Chih-Ling, 1998),

(6) $$\begin{array}{l} \text{AIC}=\frac{2K-2L}{N}+\frac{2K\left(K+1\right)}{N-K-1} \end{array}$$

where *K* = *n*_*a*_ + *n*_*b*_ + 1 was the number of parameters to be estimated, N was the length of predicted data, and $L=-\frac{N}{2}\ln\left(2\pi \right)-\frac{N}{2}\ln\left(\frac{{e_{RMSE}}^{2}}{N}\right)-\frac{N}{2}$. When *n*_*a*_ = 3 *and n*_*b*_ = 3 the valued of AIC was minimized, thus, the structure of the CAR model was

(7) $$\begin{array}{l} y_{e}\left(k\right)=-a_{1}y_{e}\left(k-1\right)-a_{2}y_{e}\left(k-2\right)-a_{3}y_{e}\left(k-3\right)\\ \;+b_{0}u\left(k\right)+b_{1}u\left(k-1\right)+b_{2}u\left(k-2\right)\\ \;+b_{3}u\left(k-3\right) \end{array}$$

and the corresponding estimated CAR model parameters in each iteration are shown in Figure 3B.

### Selection of PI Controller Parameters

Although a common Proportional-Integral-Differential (PID) controller has three control terms (P, I, and D), we only chose the P and I terms, because the D action is sensitive to the model prediction accuracy (Aström and Hägglund, 1995). With the selected CAR model, *e*_*RMSE*_ = 27.9, there were still prediction error, and the D term was not used due to these inaccuracies of the CAR model. The transformed system with the CAR model substituted for the network model was used to choose the parameters of the PI controller (Figure 1B).

The structure of a discrete PI controller was (Aström and Hägglund, 1995),

(8) $$\begin{array}{l} u\left(k\right)=u\left(k-1\right)+k_{p}\left[e\left(k\right)-e\left(k-1\right)\right]+k_{i}e\left(k\right) \end{array}$$

and the aim was to select the P term and I term coefficients, *k*_*p*_ and *k*_*i*_. The Routh-Hurwitz stability criterion (Gopal, 2002) was used to calculate automatically the PI parameters, where the selected PI controller must ensure the stability of the system. The forward transfer function of this system (Figure 1B) is given by Equation (9).

(9) $$\begin{array}{l} G\left(z\right)=\frac{Y\left(z\right)}{E\left(z\right)}=\frac{Y\left(z\right)}{U\left(z\right)}\cdot \frac{U\left(z\right)}{E\left(z\right)}=\frac{b_{0}z^{3}+b_{1}z^{2}+b_{2}z+b_{3}}{z^{3}+a_{1}z^{2}+a_{2}z+a_{3}}\cdot \frac{\left(k_{p}+k_{i}\right)z-k_{p}}{z-1}\\ \;=\frac{b_{0}\left(k_{p}+k_{i}\right)z^{4}+\left[b_{1}\left(k_{p}+k_{i}\right)-b_{0}k_{p}\right]z^{3}+\left[b_{2}\left(k_{p}+k_{i}\right)-b_{1}k_{p}\right]z^{2}+\left[b_{3}\left(k_{p}+k_{i}\right)-b_{2}k_{p}\right]z-b_{3}k_{p}}{z^{4}+\left(a_{1}-1\right)z^{3}+\left(a_{2}-a_{1}\right)z^{2}+\left(a_{3}-a_{2}\right)z-a_{3}} \end{array}$$

The closed-loop transfer function was

(10) $$\begin{array}{l} \Phi \left(z\right)=\frac{G\left(z\right)}{1+G\left(z\right)} \end{array}$$

The characteristic equation of this system was

(11) $$\begin{array}{l} D\left(z\right)=1+G\left(z\right)=\left[1+b_{0}\left(k_{p}+k_{i}\right)\right]z^{4}+[\left(a_{1}-1\right)\\ \;+\;b_{1}\left(k_{p}+k_{i}\right)-b_{0}k_{p}]z^{3}+[\left(a_{2}-a_{1}\right)+b_{2}\left(k_{p}+k_{i}\right)\\ \;\;-b_{1}k_{p}]z^{2}+[\left(a_{3}-a_{2}\right)+b_{3}\left(k_{p}+k_{i}\right)\\ \;-b_{2}k_{p}]z-a_{3}-b_{3}k_{p}=0 \end{array}$$

According to the Routh-Hurwitz stability criterion, we substituted *z* with *w*, where $z=\frac{w+1}{w-1}$, and the variable of the characteristic equation became *w*.

(12) $$\begin{array}{l} \text{D}\left(w\right)=m_{4}{\left(\frac{w+1}{w-1}\right)}^{4}+m_{3}{\left(\frac{w+1}{w-1}\right)}^{3}+m_{2}{\left(\frac{w+1}{w-1}\right)}^{2}\\ \;+m_{1}\left(\frac{w+1}{w-1}\right)+m_{0}=0 \end{array}$$

Combining Equations (11) and (12), *m*_4_ = 1+*b*_0_(*k*_*p*_+*k*_*i*_), *m*_3_ = (_*a*_1_ − 1)+*b*1_(*k*_*p*_+*k*_*i*_) − *b*_0_*k*_*p*_, *m*_2_ = (*a*_2_ − *a*_1_)+*b*_2_(*k*_*p*_+*k*_*i*_)−*b*_1_*k*_*p*_, *m*_1_ = (_*a*_3_ − *a*_2_)+*b*3_(*k*_*p*_+*k*_*i*_)−*b*_2_*k*_*p*_, *m*_0_ = −*a*_3_ − *b*_3_*k*_*p*_. Then multiplying both sides of Equation (12) by (*w*−1)^4^, such that, ${\left(w-1\right)}^{4}D\left(w\right)=n_{4}w^{4}+n_{3}w^{3}+n_{2}w^{2}+n_{1}w+n_{0}=0$, that is,

(13) $$\begin{array}{l} D_{1}\left(w\right)=n_{4}w^{4}+n_{3}w^{3}+n_{2}w^{2}+n_{1}w+n_{0}=0 \end{array}$$

where *n*_4_ = *m*_0_+*m*_1_+*m*_2_+*m*_3_+*m*_4_, *n*_3_ = −4*m*_0_−2*m*_1_+2*m*_3_+4*m*_4_, *n*_2_ = 6*m*_0_−2*m*_2_+6*m*_4_, *n*_1_ = −4*m*_0_ + 2*m*_1_ − 2*m*_3_ + 4*m*_4_, *n*_0_ = *m*_0_ − *m*_1_ + *m*_2_ − *m*_3_ + *m*_4_.

The stability of this system was equivalent to the following conditions:

(14) $$\begin{array}{l} n_{i}>0\left(i=0,1,2,3,4\right),n_{3}n_{2}>n_{4}n_{1},n_{3}n_{2}n_{1}>n_{4}n_{1}^{2}+n_{3}^{2}n_{0} \end{array}$$

Combining Equations (11)–(13), *n*_*i*_could also be described as a function of *k*_*p*_ and *k*_*i*_, and to ensure that all conditions in Equation (14) were satisfied, we chose *k*_*p*_ = 0.80, *k*_*i*_ = 0.05.

### Closed-Loop Frequency Modulation

Considering the established physiological responses to different pulse repetition frequencies of DBS (Birdno and Grill, 2008), we constrained the calculated stimulation frequency to between 5 and 200 Hz. When the calculated frequency was larger than 200 Hz, it was set to 200 Hz; when the calculated frequency was <5 Hz, it was set to 5 Hz.

(15) $$u\left(k\right)=\{\begin{array}{cc} 5 & u\left(k\right)<5\\ u\left(k-1\right)+k_{p}\left[e\left(k\right)-e\left(k-1\right)\right]+k_{i}e\left(k\right)\; & 5\leq u\left(k\right)\leq 200\\ 200 & u\left(k\right)>200 \end{array}$$

The stimulation frequency was calculated using the PI controller, which required knowledge of the beta power at the k*th* and (k-1)*th* time points. The beta power of the k*th* time point was calculated from (t−0.1) s to t s, the beta power of the (k-1)*th* time point was calculated from (t_1_-0.1) s to t_1_ s. The time difference between t and t_1_ was 0.008 s. Note this was not the time step for the controller to update the DBS frequency, and the controller updated the DBS frequency only after the former interpulse interval ended.

## Results

### Prediction Performance of the CAR Model

**Figure 4:**
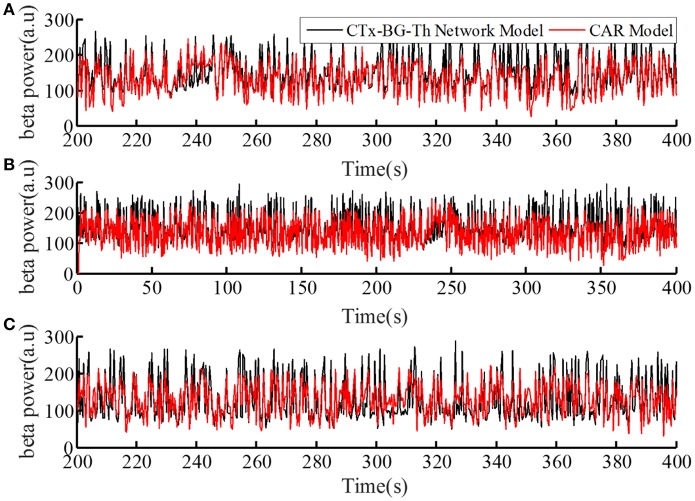
The prediction performance of the CAR model during model training **(A)** and testing **(B,C)**. The datasets used to train and test the CAR model were generated as described in section Computational Model of the Cortex-Basal Ganglia-Thalamus Network. The black line represented the beta power calculated from the original CTx-BG-Th network model, and the red line represented the beta power data predicted by the identified CAR model.

The performance of the CAR model during the model training process is shown in Figure 4A. The correlation coefficient between the actual and estimated data in the model training process was *r*(*y, y*_*e*_) = 0.84. In addition, we generated different sequences of random stimulation frequencies, and delivered the corresponding stimulation signals to the network model to calculate the resulting sequences of beta power. The same sequences of stimulation frequencies were also delivered to the trained CAR model. The prediction performance of the trained CAR model on two example data sequences is shown in Figures 4B,C. In this testing phase, the correlation coefficient between the two outputs were *r*(*y, y*_*e*_) = 0.82 and *r*(*y, y*_*e*_) = 0.80. Thus, the prediction accuracy of the CAR model was ~80%.

To create a quantitative comparator for the prediction accuracy of the identified CAR model, we delivered an identical test stimulation signal to the CTx-BG-Th network model five times. The mean correlation coefficient among any two output datasets was 0.95. Since the CTx-BG-Th was highly non-linear, while the structure of the CAR model presented here was linear, the difference between 0.95 and 0.8 may reflect the unmodeled non-linear dynamics between the stimulation frequency and the beta power. However, since our aim in identifying the CAR model was as a tool to design the PI controller, the 80% accuracy was deemed sufficient.

### Tracking of Constant Beta Power

**Figure 5:**
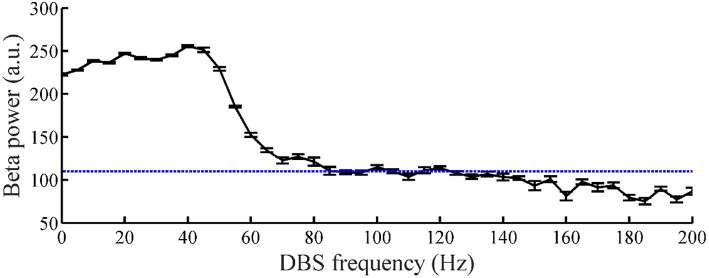
The relationship between DBS frequency and the beta band power of GPi model neuron spike times. Standard error bars are shown for 50 trials. The dotted line labels the 110 target beta power value.

The relationship between the DBS pulse repetition frequency and the beta power of GPi model neuron spike times in the CTx-BG-Th model is shown in Figure 5. The beta band power in the healthy and PD states of the CTx-BG-Th model were 162 and 222.5, respectively. Similar to the effects of DBS frequency on motor symptoms (Birdno and Grill, 2008), reductions in beta band oscillatory activity were observed only for higher frequencies of DBS. The target beta power was selected to be 110, which was approximately the value generated by DBS at 115 Hz. When the stimulation frequency was larger than 100 Hz, the variations of beta power with changes in frequency were quite small, and the selection of a specific beta power target level had no particular impact on the results.

**Figure 6:**
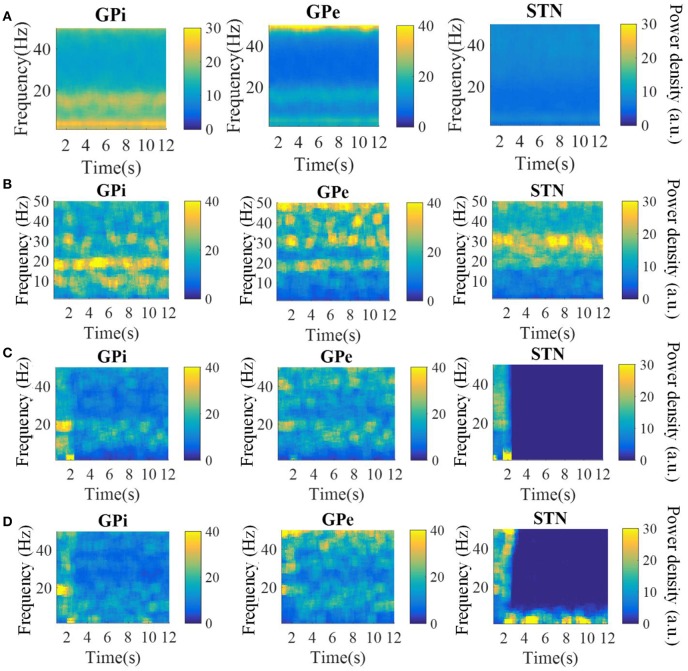
Spectrograms of the spike times from model GPi, GPe, and STN neurons in the normal **(A)**, parkinsonian condition **(B)**, during 115 Hz DBS **(C)**, and during CL DBS **(D)**. In the parkinsonian condition, all neurons exhibited excessive oscillatory activity compared with the normal condition. The 115 Hz DBS and CL DBS began at *t* = 2 s, and greatly reduced the beta band oscillatory activity.

**Figure 7:**
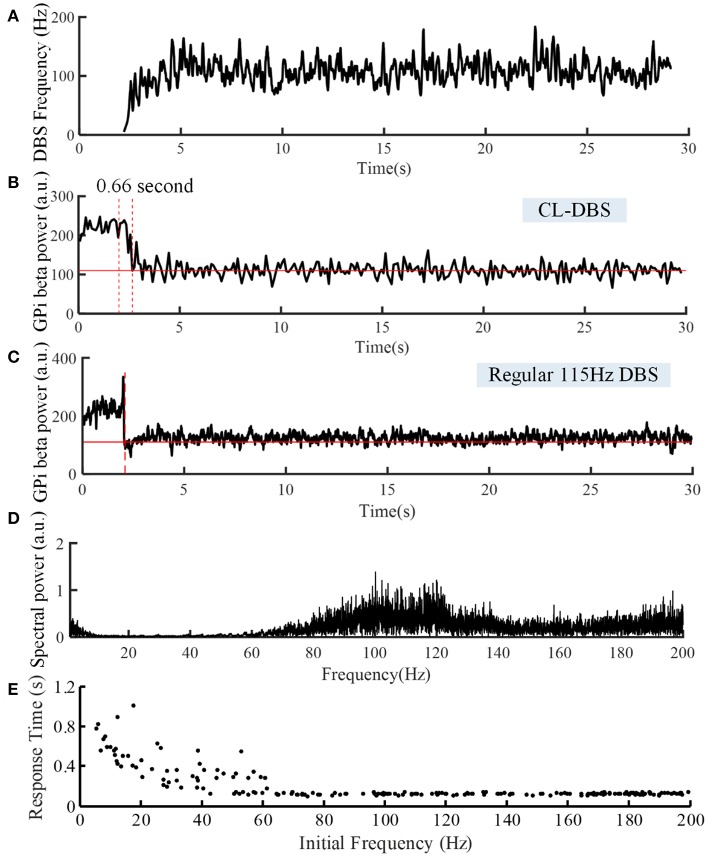
Variations of DBS frequency **(A)** and beta power of spike times from model GPi neurons during CL DBS **(B)** and regular 115 Hz DBS **(C)**. The stimulation signal began at *t* = 2 s, and the beta power converged to the target of 110 at *t* = 2.66 s in **(B)** and at *t* = 2.09 s in **(C)**. **(D)** The corresponding spectral power of the stimulation sequence for this CL DBS example. **(E)** The relationship between the initial frequency of the CL controller and response time in the CL DBS system. The initial frequency of CL DBS was randomly selected from a uniform distribution between 5 and 200 Hz, and the corresponding response time was calculated across 200 trials.

The spectrograms of the spike times from model GPi, GPe, and STN neurons in the parkinsonian condition, during 115 Hz DBS, and during CL DBS are shown in Figure 6. Under the parkinsonian condition, the model neurons in these three nuclei exhibited oscillatory activity around 20 Hz. During the 115 Hz DBS and CL DBS cases, stimulation began at *t* = 2 s, after which the oscillatory activity rapidly diminished. CL DBS produced intermittent oscillatory activity in model STN neurons in the low frequency band (3–12 Hz), which was 6.2 times larger than the lower frequency power present during open loop DBS (OL DBS) at 115 Hz. The dynamic sequence of stimulation frequencies during CL DBS (Figure 7A) exhibited peaks in the power spectrum both around 115 Hz and between 3 and 12 Hz. DBS (Figure 7D). The stimulation signal power 3–12 Hz generated oscillatory activity in model STN neurons in the low frequency band that was larger than during 115 Hz OL DBS. Thus, although both stimulation methods reduced the power in the beta band, they may act through different mechanisms.

The variations of DBS frequency and the corresponding changes in beta band power in model GPi neurons during CL DBS are shown in Figures 7A,B, respectively. The stimulation began at *t* = 2 s, the initial stimulation frequency was set to 5 Hz, and the CL DBS system calculated the subsequent frequencies automatically to drive the beta band power to the target of 110. The mean stimulation frequency from 2 to 30 s was 118.7 Hz, and the mean beta power from 2 to 30 s was 114.3, while the mean beta power during OL DBS from 2 to 30 s was 111.3 (Figure 7C). Compared to OL DBS at 115 Hz, the CL DBS controller generated a wider distribution of power in the stimulation frequency sequence (Figure 7D), and the power present in the low frequency band of the stimulation signal generated low frequency power in STN model neurons during CL DBS (Figure 6D). The response time was shorter for open loop 115 Hz DBS (0.09 s) than for CL DBS (0.66 s); however, the response time was strongly dependent on the initial value of frequency during CL DBS (Figure 7E). As the initial frequency was increased the response time decreased, and when the initial frequency was ≥60 Hz, the response time for CL DBS was <0.15 s.

**Figure 8:**
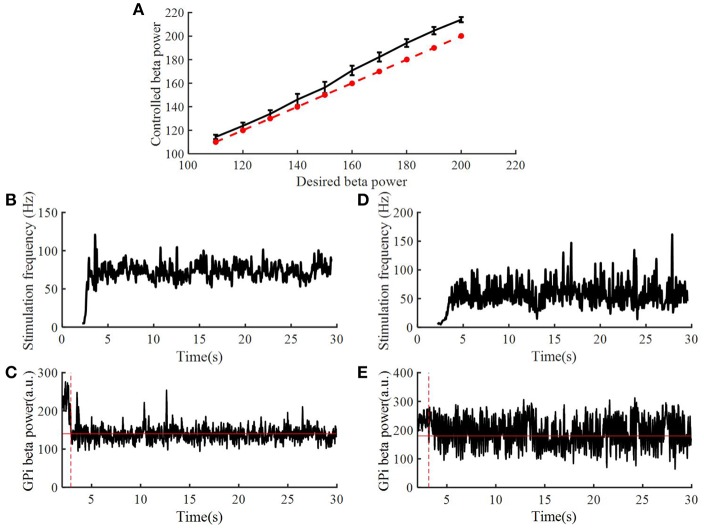
Performance of the PI controller across different levels of target beta power. **(A)** The dotted line represented the value of desired beta power, and the solid line represented the value of controlled beta power; standard error bars are shown for 50 trials. The variation of DBS frequency and beta power of model GPi neuron spike times when the desired beta power was 140 **(B,C)** and 180 **(D,E)**, respectively. The red solid line in **(C,E)** are the desired beta power value. The red dotted line in **(C,E)** indicate the time when the controlled beta power reached the desired beta power.

To assess the robustness of the PI controller, we changed the target beta power while keeping the PI parameters unchanged (Figure 8A). Figures 8B–E illustrate the stimulation frequency and beta power variation when the desired beta power was 140 and 180, respectively. When the target beta power was 140, the response time was 0.89 s, and the mean stimulation frequency was 74 Hz. When the target beta power was 150, the response time was 1.15 s, and the mean stimulation frequency was 56 Hz. When the target beta power was larger than 160, the tracking performance declined. Thus, as the desired beta power was larger, the convergence time of GPi beta power became longer. When the target beta power was set to 60 (i.e., a value not achievable with OL DBS, Figure 5), the calculated stimulation frequency varied between 155 and 200 Hz (mean = 177.8 Hz), the mean beta power from 2 to 30 s was 82.3, and with OL DBS at 177.8 Hz, the mean beta power was 87.91.

### Tracking of Dynamic Changes in Target Beta Power

Beta power in the BG exhibits dynamic changes prior to and during voluntary movement and a fixed target beta power may not be appropriate for functional control of DBS.

**Figure 9:**
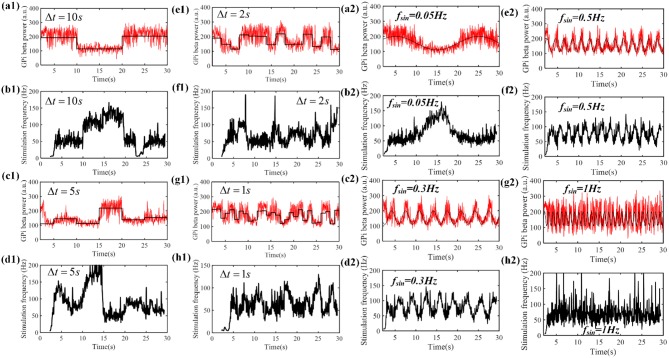
**(a1–h1)** The performance of the PI controller tracking dynamic changes in target beta power and the corresponding variation in stimulation frequency by the CL DBS system. The duration of the target beta power was 10 s **(a1)**, 5 s **(c1)**, 2 s **(e1)** and 1 s **(g1)**, respectively. The black line represents the desired beta power, and the red line represents the actual beta power during CL DBS. **(b1,d1,f1,h1)** show the respective variations in stimulation frequency during CL DBS. When the duration of target beta power declined from 10 to 0.5 s, the correlation coefficients between the desired and actual beta power were 0.83, 0.82, 0.71, 0.69, 0.49, respectively. **(a2–h2)** The performance of the PI controller tracking sinusoidal trajectories at different frequencies and the associated DBS frequencies determined by the CL controller. The black line in **(a2,c2,e2,g2)** represented the desired beta power, and the red line represented the actual beta power. When the frequency of target sinusoidal trajectories increased from 0.05 to 1 Hz, the correlation coefficients between the desired and actual beta power were 0.85, 0.65, 0.49, and 0.17, respectively. Sinusoidal trajectories of target beta power with frequencies ranging from 0.05 to 1 Hz were also tested. The BG model can generate beta power between 90 and 200 during regular DBS, and the minimum and maximum amplitude of the target sinusoidal trajectories were therefore set to be 90 and 200, respectively. The tracking performance and variation of stimulation frequency of the CL DBS system are shown in **(a2–h2)**, and the correlation coefficient between the actual beta power and the target trajectory was used to quantify the tracking accuracy. The tracking performance declined with the increase in target sinusoidal frequency, and the correlation coefficient was 0.85, 0.65, 0.49, and 0.17 for sinusoidal frequencies of 0.05, 0.3, 0.5, and 1 Hz, respectively.

Therefore, we tested the performance of the control system with time-varying beta power. According to Figure 5, when the stimulation frequency of regular DBS increased from 50 to 130 Hz, the GPi beta band power decreased gradually from 220 to 110, and the beta band power tended to saturate at DBS frequencies larger than 130 Hz. Therefore, the target values randomly selected from a uniform distribution between 110 and 220. The duration of the target value varied from 10 to 1 s (Figures 9a1–h1). The correlation coefficient between the target beta power and actual beta power between 3.5 and 30 s was calculated, as this mitigated the confounding effects of the initial stimulation frequency. The tracking performance of the CL DBS declined with the duration of the target value, and when the duration was 10, 5, 2, 1, and 0.5 s, the correlation coefficients were 0.83, 0.82, 0.71, 0.69, and 0.49, respectively. Sinusoidal trajectories of target beta power with frequencies ranging from 0.05 to 1 Hz were also tested. The BG model can generate beta power between 90 and 200 during regular DBS, and the minimum and maximum amplitude of the target sinusoidal trajectories were therefore set to be 90 and 200, respectively. The tracking performance and variation of stimulation frequency of the CL DBS system are shown in Figures 9a2–h2, and the correlation coefficient between the actual beta power and the target trajectory was used to quantify the tracking accuracy. The tracking performance declined with the increase in target sinusoidal frequency, and the correlation coefficient was 0.85, 0.65, 0.49, and 0.17 for sinusoidal frequencies of 0.05, 0.3, 0.5, and 1 Hz, respectively.

## Discussion

Beta band oscillatory activity in the BG is correlated with motor symptoms in PD and may be a suitable biomarker for CL DBS in PD (Little and Brown, 2012; Hoang et al., 2017). For example, Arlotti et al. (2016b) and Little et al. (2013) used the beta oscillation amplitude to control the on time of DBS. DBS was delivered only when the beta-band oscillation amplitude was larger than a pre-set threshold, which reduced energy consumption compared to continuous DBS, while increasing the therapeutic effects on motor symptoms. Subsequently, Dan et al. demonstrated that this approach was also effective in a PD patient with chronically implanted DBS (Piña-Fuentes et al., 2017). In complementary modeling studies, Grant and Lowery designed a CL DBS system to modulate the amplitude of DBS based on beta band oscillations of LFPs, where the coupling strength within the cortico-basal ganglia network was altered to illustrate the ability of CL DBS to respond to changes in network activity (Grant and Lowery, 2013).

However, beta oscillatory activity exhibits dynamic changes (desynchronization) during movement, and Johnson et al. found that a constant beta set point may not be suitable as CL DBS performed poorly during reaching behavior (Johnson et al., 2016). Therefore, if beta power is to be used as a feedback control signal, a constant reference value might not be appropriate. In more recent studies, DBS voltage was adjusted proportionally to the STN LFP beta power, and this adaptive DBS reduced side effects compared to traditional open-loop DBS (Rosa et al., 2015; Arlotti et al., 2018). In another alternative to simply reducing oscillatory activity below a fixed threshold, Santaniello et al. automatically adjusted the stimulation voltage in a mathematical model to match a desired profile of oscillatory neuronal activity (Santaniello et al., 2011). During go/no-go voluntary movements, dynamic changes in beta band power occur at 0.3–1Hz (Sanes and Donoghue, 1993; Zaepffel et al., 2013). The proposed controller could track dynamic changes slower than 1 Hz, and thus such an approach may account for the dynamic changes in beta oscillatory power that occur during movement. Instead of simply switching the stimulation on and off, or adjusting the stimulation amplitude, the controller regulated the stimulation frequency in real time. If the variation in beta band power during a wide range of movements was known a priori, such a closed-loop system that modulates stimulation frequency to track dynamic beta oscillatory activity may facilitate a wide range of individual patient motor behaviors.

The proposed closed-loop stimulation algorithm was simulated using a validated CTx-BG-Th model (Kumaravelu et al., 2016). There are several other potential models of the network effects of DBS, which might be used for development and evaluation of closed-loop controllers. Hahn and McIntyre developed a network model of the effects of DBS in the STN of the parkinsonian non-human primate, and demonstrated that effective DBS suppressed burst activity in the GPi (Hahn and McIntyre, 2010). Subsequently, Holt and Netoff implemented a mean field version of this model and analyzed the effects of different frequencies of DBS (Holt and Netoff, 2014). Similarly, Santaniello et al. (2015) implemented a network model of the effects of STN DBS in the parkinsonian non-human primate and demonstrated the importance of both antidromic and orthodromic activation. We selected the Kumaravelu et al. network model because it replicated a wide range of electrophysiological data from the unilateral 6-OHDA lesioned rat model of PD (Kumaravelu et al., 2016) thereby facilitating subsequent *in vivo* evaluation of the controller.

The proposed CL DBS controller was successful at regulating the beta oscillatory activity of spike times of model GPi neurons to track different beta reference values. The stimulation frequency was automatically calculated by the PI controller, and PI parameters were calculated using stability analysis of the system rather than trial-and-error adjustment (Gorzelic et al., 2013). However, there were several potential limitations of the proposed CL DBS method. The identified linear CAR model described only 80% of the relationship between the stimulation frequency and the beta power. Therefore, although the PI controller was robust to changes in the reference beta power, the dynamic changes in beta power could be tracked well only at frequencies of ≤ 1 Hz. When the target beta power changed faster than 1 Hz, the tracking error increased, likely as a result of the unmodeled dynamics. In subsequent trial-and-error tuning, it appeared that the best PI controller parameters were different for different beta power targets. Thus, adaptive controllers that modulate the PI controller parameters with the variation of target beta power may improve the tracking performance for dynamic reference signals. The CTx-BG-Th network was highly non-linear, and performance might also be improved using a non-linear controller. The beta oscillatory power was selected as the biomarker in this study, however, other biomarkers such as the spike time entropy (Dorval et al., 2008) and phase amplitude coupling (de Hemptinne et al., 2015) are also correlated with parkinsonian symptoms, and might be suitable feedback control signals. The application of other biomarkers or multiple biomarkers in the design of closed-loop stimulation for PD is worth exploring (Hoang et al., 2017). The controller regulated the stimulation frequency, but the effects of DBS are also dependent on the pulse amplitude, pulse duration, and stimulation pattern (Kuncel and Grill, 2004; Grill, 2018). Further, Holt et al. demonstrated that the effects of burst DBS in a network model of the basal ganglia (Hahn and McIntyre, 2010) were strongly dependent on timing relative to the phase of oscillatory activity (Holt et al., 2016).

We demonstrated successful tracking of different dynamic beta power reference signals, and the simulated dynamic targets could represent different movements of PD patients. Thus, an important challenge to implement the proposed CL DBS approach experimentally or clinically is to determine the relationship between reference beta oscillation power and the movement. In addition to real-time electrophysiological recording, movement sensors might also be useful to establish the dynamic reference signal.

## Conclusion

CL DBS was proposed to reduce energy consumption and alleviate side effects compared to continuous fixed-parameter DBS. This requires design of a suitable closed-loop system that can account for dynamic changes in the feedback signal that occur during voluntary movement. We used the beta oscillatory power of GPi model neuron spike times as a biomarker of model state, and used a PI controller to calculate the DBS frequency according to dynamic variations in the beta power. This closed-loop adjustment of stimulation frequency approach was tested in a computational model of the CTx-BG-Th network and was able to track constant as well as dynamic beta oscillatory activity.

## Data Availability

The raw data supporting the conclusions of this manuscript will be made available by the authors, without undue reservation, to any qualified researcher.

## Author Contributions

All authors listed have made a substantial, direct and intellectual contribution to the work, and approved it for publication.

### Conflict of Interest Statement

The authors declare that the research was conducted in the absence of any commercial or financial relationships that could be construed as a potential conflict of interest.
